# Pilot study of newborn screening for six lysosomal storage diseases using Tandem Mass Spectrometry^☆^

**DOI:** 10.1016/j.ymgme.2016.05.015

**Published:** 2016-05-20

**Authors:** Susan Elliott, Norman Buroker, Jason J. Cournoyer, Anna M. Potier, Joseph D. Trometer, Carole Elbin, Mack J. Schermer, Jaana Kantola, Aaron Boyce, Frantisek Turecek, Michael H. Gelb, C. Ronald Scott

**Affiliations:** a Department of Pediatrics, University of Washington, Seattle, WA 98195, United States; b PerkinElmer, Waltham, MA 02451, United States; c Chemistry, University of Washington, Seattle, WA 98195, United States; d Biochemistry, University of Washington, Seattle, WA 98195, United States; e PerkinElmer, Turku 20750, Finland

**Keywords:** Newborn screening, Lysosomal storage disorders, Gaucher disease, Krabbe disease, Hurler disease, Pompe disease, Fabry disease, Niemann-Pick-A/B disease, Tandem mass spectrometry, Dried blood spot

## Abstract

**Background:**

There is current expansion of newborn screening (NBS) programs to include lysosomal storage disorders because of the availability of treatments that produce an optimal clinical outcome when started early in life.

**Objective:**

To evaluate the performance of a multiplex-tandem mass spectrometry (MS/MS) enzymatic activity assay of 6 lysosomal enzymes in a NBS laboratory for the identification of newborns at risk for developing Pompe, Mucopolysaccharidosis-I (MPS-I), Fabry, Gaucher, Niemann Pick-A/B, and Krabbe diseases.

**Methods and Results:**

Enzyme activities (acid α-glucosidase (GAA), galactocerebrosidase (GALC), glucocerebrosidase (GBA), α-galactosidase A (GLA), α-iduronidase (IDUA) and sphingomyeline phosphodiesterase-1 (SMPD-1)) were measured on ~43,000 de-identified dried blood spot (DBS) punches, and screen positive samples were submitted for DNA sequencing to obtain genotype confirmation of disease risk. The 6-plex assay was efficiently performed in the Washington state NBS laboratory by a single laboratory technician at the bench using a single MS/MS instrument. The number of screen positive samples per 100,000 newborns were as follows: GAA (4.5), IDUA (13.6), GLA (18.2), SMPD1 (11.4), GBA (6.8), and GALC (25.0).

**Discussion:**

A 6-plex MS/MS assay for 6 lysosomal enzymes can be successfully performed in a NBS laboratory. The analytical ranges (enzyme-dependent assay response for the quality control HIGH sample divided by that for all enzyme-independent processes) for the 6-enzymes with the MS/MS is 5- to 15-fold higher than comparable fluorimetric assays using 4-methylumbelliferyl substrates. The rate of screen positive detection is consistently lower for the MS/MS assay compared to the fluorimetric assay using a digital microfluidics platform.

## 1. Introduction

There is widespread interest in expanding newborn screening (NBS) panels to include those lysosomal storage diseases for which initiation of treatment leads to a more optimal clinical outcome. The Secretary of Health and Human Services in the USA has recommended that Pompe and mucopolysaccharidosis-I (MPS-I) diseases be added to the Recommended Uniform Screening Panel.⋯Some states in the USA and some additional countries are screening for up to 7 lysosomal storage diseases.⋯For example, IL, MO, NY and Taiwan have live NBS programs for one or more LSDs. Several states including CO, IA, MA, MI, NE, NJ, NM, OH, PA, TN, TX, and WI are making steps towards implementation of LSD NBS (https://www.newsteps.org).

Chamoles and co-workers were the first to demonstrate that several lysosomal enzymes retain their enzymatic activities in rehydrated punches from dried blood spots on NBS cards (DBS) [1–6], which set the stage for NBS based on direct enzymatic activity assay. Our laboratory has extensively developed the use of tandem mass spectrometry (MS/MS) for the quantification of lysosomal enzyme activities in DBS [7,8]. Some lysosomal enzymes can also be assayed using fluorimetric methods (see references above by Chamoles et al). A comparison of NBS methods based on lysosomal enzymatic activity assay (including MS/MS and fluorimetry), lysosomal protein abundance assays, lysosomal biomarker assays, and lysosomal enzyme gene sequencing has been reviewed [9]. Enzymatic activity-based NBS is the furthest along in terms of re-liability, reagent development and use in NBS laboratories.

We completed the first large-scale (n ~ 100,000) pilot study of 3 lysosomal storage diseases, Fabry, MPS-I, and Pompe, using flow injection-MS/MS [10] that showed the feasibility of this method for NBS. This study was performed with our original enzyme substrates [7,8], which have been made available worldwide by a collaboration between Genzyme Pharmaceuticals and the Centers for Disease Control and Prevention along with quality control DBS [11]. In the past few years, we have further optimized the multiplex MS/MS method by making the following improvements: 1) Elimination of the solid-phase extraction step prior to MS/MS; 2) Use of internal standards which are chemically identical to the enzymatically-generated products but differentiated by deuteration; 3) Development of a buffer that supports incubation for analysis of 6 lysosomal enzymes in a single mixture; 4) Modification of the ceramide-based substrates to improve solubility in assay cocktail; 5) Development of a new MPS-I substrate that improves ionization of the product in the mass spectrometer [12]. In this way we developed a new 6-plex, flow injection-MS/MS assay for Fabry, Gaucher, Krabbe, MPS-I, Niemann-Pick-A/B, and Pompe diseases. In the current study, we report the results of a pilot study of the new 6-plex assay in the WA state NBS laboratory performed on ~43,000 newborn DBS. The patient samples were de-identified, but we provide genotype data on screen-positive samples to provide an estimate of the number of affected individuals.

## 2. Methods

### 2.1. Materials

All studies with human subject samples were carried out by Institutional Review Board approval from the Washington state (IRBB-062707-H). De-identified DBS were leftover material after routine NBS. They were stored at ambient temperature and exposed to air for 30–60 days prior to punching into 96-well plates for the current study. Three punches were taken per DBS; one was submitted for the 6-plex assay, and the other two were held for potential genotyping (typically only 1 punch needed for genotyping). Quality control DBS were obtained from the Centers for Disease Control and Prevention or from PerkinElmer.

### 2.2. 6-Plex assay

A fully detailed standard operating procedure for the 6-plex assay is given in Data in Brief. Flow injection-MS/MS was performed on a Waters Acquity TQD Ultra Performance instrument (settings in Data in Brief for this and other instruments). The Waters brand QuanLynx software package was used to integrate all product and internal standard multiple reaction monitoring (MRM) peaks. Performance stability of the 6-plex assay was evaluated on a regular basis using the quality control DBS, and methodology and results are provided in Data in Brief.

### 2.3. Genotyping

Genotypes were determined as previously described [10]. The University of Washington genome sequence variation database [13,14] and the ExAC server (http://exac.broadinstitute.org) were used to classify variants as previously known, extremely rare, or exclusive to the patient tested. Rare DNA variants were also used as search queries to check for publications containing disease-associated mutations.

## 3. Results

### 3.1. Description of the 6-plex assay

**Fig. 1:**
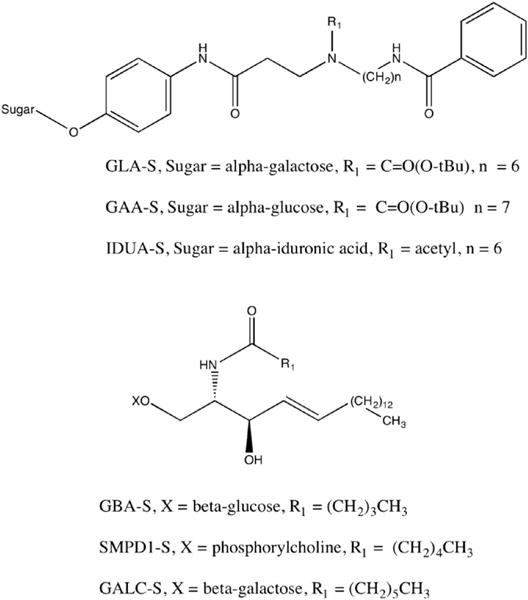
Substrates used for the 6-plex assay. For GLA, GAA, and IDUA the enzymatic products are the substrates with the sugar replaced with hydrogen, and the internal standards are the products with 5 deuteriums on the benzoyl group. For GBA, GALC and SMPD1, the product is the substrate with the sugar (GBA and GALC) or the phosphocholine (SMPD1) replaced with hydrogen. The internal standards are the products but with deuterium in the fatty acyl chain (CD_3_CD_2_CD_2_ for SMPD1, and CD_3_CD_2_ for GALC) or in the sphingosine chain (CD_3_CD_2_CD_2_ for GBA).

Fig. 1 shows the set of 6 lysosomal enzyme substrates used in the new 6-plex assay. Substrates for sphingomyelin phosphodiesterase-1 (SMPD1-S), α-galactosidase A (GLA-S), and acid α-glucosidase (GAA-S) are identical to those developed previously [7,8] and distributed by the Centers for Disease Control and Prevention. The original substrate for glucocerebrosidase (GBA-S) had a 12-carbon fatty acyl chain on the sphingosine. This has now been shortened to a 5-carbon fatty acyl chain to improve rapid dissolution in assay cocktail. Likewise, our original galactocerebrosidase (GALC) substrate contained an 8-carbon fatty acyl chain and now has a 7-carbon chain. Studies (not shown) indicated that the GALC enzyme, but not the glucocerebrosidase (GBA) enzyme, displays a ~5-fold decrease of enzymatic activity when the fatty acyl chain is shortened to 5 carbons, hence our decision to incorporate the shortest fatty acyl chain into the GBA substrate. The aglycone of our original α-iduronidase (IDUA) substrate has been substantially modified leading to an improvement of ionization in the electrospray source of the MS/MS [12]. Reasons for this improved detection sensitivity have been proposed [15].

The measured enzymatic products are the substrates lacking the sugar group (or phosphocholine in the case of SMPD1). The internal standards are chemically identical to the products but differentiated with deuteration (sites of deuteration given in Fig. 1).

The second improvement was to optimize the assay buffer from the original compositions [7,8,16] such that all 6 enzymes could be assayed using a single cocktail without a significant decrease in enzymatic activity.⋯Sodium taurocholate was used to solubilize the sphingolipids for presentation to SMPD1, GALC, and GBA. Sodium oleate was added as it increases the activity of the enzymes that operate on sphingolipids. Sodium oleate is a solid, and this is less prone to oxidation during storage than liquid oleic acid. The sodium salt also dissolves more rapidly during buffer preparation. Zinc chloride was added as an activator of SMPD1. Acarbose was used as a selective inhibitor of maltase glucoamylase, which would otherwise partially hydrolyze the acid α-glucosidase (GAA) substrate [7]. *D*-Saccharic acid 1,4-lactone was used as an inhibitor of β-glucuronidase to suppress any product formation from trace β-glycoside present in IDUA-S (difficult to completely eliminate since IDUA-S is made by isomeration of the glucuronide intermediate). *N*-Acetylgalactosamine was added to block α-galactosidase B [17, 18], which would otherwise hydrolyze the substrate for α-galactosidase A (GLA-S). We screened a variety of buffers in the pH range of 3.5 to 6.5 to find conditions for high activity of all 6 enzymes. Succinic acid emerged as the best candidate.⋯Additional details for buffer optimization are given in Supplemental material.

The last improvement was in response to concern from some NBS laboratories about the solid-phase extraction step in the original assay. Although the step is simple to execute with 96-well filter plates and a vacuum manifold, the dry down step with >1 mL of eluant solvent added 1–2 h to the process. We have now replaced the solid-phase extraction step with liquid–liquid extraction with a minimal volume of ethyl acetate. This step serves a few purposes: 1) Buffer and blood salts, blood proteins, and the majority of the sodium taurocholate detergent remain in the water layer, which minimizes suppression of ionization during electrospray ionization and source dirtying; 2) A substantial fraction of the uncleaved substrates remain in the water layer, and this helps to minimize background signal due to cleavage of the substrates in the heated electrospray ionization source to give background product signals. Neither solid-phase extraction nor liquid-liquid extraction is needed if liquid chromatography is used coupled to MS/MS [19,20], but most NBS centers are not equipped for liquid chromatography with binary solvent gradients.

The workflow for the 6-plex assay is summarized as follows starting from plates of punched DBS (all steps carried out by a single laboratory worker at the bench). All liquid transfers were done with a single 96-channel, manually operated pipette (Rainin Liquidator). On day 1, a single assay cocktail containing buffer, 6 substrates and 6 internal standards was added to the plate of DBS punches. The plate was sealed and placed in the temperature-controlled incubator/shaker for overnight incubation (addition of cocktail to the plates takes ~15 min on day 1). On day 2, the reactions were quenched with ethyl acetate/methanol and submitted to liquid-liquid extraction after addition of ethyl acetate (minimal toxicity) and additional water. After a brief centrifugation to separate the solvent layers, a portion of the upper organic layer was transferred to a new plate, and solvent was evaporated with jets of air. After reconstitution in flow injection solvent, the plates were ready to be placed on the autosampler of the MS/MS instrument for flow injection-MS/MS (sample preparation takes ~3 h for 6 plates on day 2). The sample-to-sample inject time on the MS/MS instrument was 115 s, thus 657 samples (3,942 assays) (6.8 plates, 544 newborns) can be analysed from 12 pm on day 2 to 9 am of day 3, after which they are ready for review by a lead worker. This corresponds to 113,152 newborns per year per MS/MS instrument based on 4 rounds of as-says per week.

#### 3.2. 6-Plex study results

To establish our first round of screen cutoffs, we made use of our previous study data on a 3-plex MS/MS assay for Pompe, Fabry and MPS-I [7]. To NBS lab [21]. The variation of the daily mean activity for each of the 6 enzymes is given in Data in Brief.⋯Each 96-well sample plate also contains 16 quality control DBS (plate layout given in Data in Brief). We anticipate reducing the number of quality control wells to 8 since there is no need in the future to run quality controls from both PerkinElmer and the Centers for Disease Control and Prevention. This will increase the number of newborns analyzed per year per MS/MS instrument from 113,152 to 124,467.

Table 1 gives the pilot study results for ~ 43,000 de-identified newborn DBS. For each of the 6 enzymes we give the screen cutoff, the number of screen positives (those at or below the cutoff), and the enzymatic activity (% of daily mean and absolute value) of each screen positive sample. For each screen positive, we retrieved the duplicate DBS from the same specimen and sequenced the relevant enzyme gene (all exons and exon-intron boundaries). The genotype for each screen positive is given in Table 1 along with references. Table 5 of Data in Brief gives the typical peak areas (from integration of the ion chromatograms) for each of the 6 internal standards along with typical peak areas for the enzymatic products from a random newborn. All values are in the range of 100,000–600,000 ion counts. In the same table is also given the product peak areas for each of the 6 blanks in which a 3 mm punch of filter paper (no blood) was incubated and processed as for the DBS assays (values in the range 1100–6300).

For IDUA (MPS-I), the mean activity was 6.56 μmol/h/L. With a screen cutoff of 10% of the daily mean, we obtained a total of 6 screen-positive samples. After genotyping we found two gene carriers with enzyme activity at 4% of the daily mean. Each of these two samples contained a single missense mutation. Four samples contained DNA changes that have been identified as low activity variants that are common in the African-American population (ExAC server).

For GAA (Pompe), the mean activity was 12.41 μmole/h/L, and 2 samples displayed activity below the cutoff of 10% of daily mean. One sample was shown to have non-pathogenic variants common in the Asian population [22]. The lowest activity samples gave a genotype with a novel complex rearrangement of the *GAA* gene resulting in homozygosity for the frameshift p.A724Gfs^*^44, and this leads to a nearby stop codon. We anticipate that this patient will be affected with Pompe disease.

For GLA (Fabry), the mean activity was 17.33 μmole/h/L, and 8 samples were below the cutoff of 18% of the daily mean activity. Two had the normal *GLA* gene sequence, two had benign variants, including the common p.A143T, and three were determined to be affected. Two of these had the amino acid change, p.K213N, which occurs at the 3′ end of exon 4 and disrupts the splice site. The third, p.R356Q, is predicted to be pathogenic based on a prior confirmed clinical case.⋯These findings suggest the frequency of Fabry disease approaches 1 in ~10,000 male births.

For GBA (Gaucher), the mean activity was 12.69 μmole/h/L. Three were screen positive using a cutoff of 10% of the daily mean activity. One was found to have the normal *GBA* gene sequence. One sample was found to be a carrier for the common mutation p.N370S, and one sample is categorized as probably affected with Gaucher disease type I based on prior studies (Table 1).

For SMPD1 (Niemann–Pick–A/B), the mean activity was 6.03 μmole/h/L. Five samples dispalyed enzymatic activity below the cutoff of 20% of the daily mean activity. One sample gave a normal sequence for the *SMPD1* gene. One sample contained a single nucleotide missense alteration, and two contained p.G508R, a common population variant (20% in the northern European population) known to be non-pathogenic. One sample is strongly predicted to be affected, being homozygous for a stop codon early in the coding sequence. Thus, the estimated frequency is 1 in 44,000 for Niemann–Pick–A/B.

For GALC (Krabbe), the mean activity was 5.04 μmol/h/L. With a screen cutoff of 9.6%, 11 samples displayed low activity below the cutoff. None of the samples had nucleotide changes consistent with a diagnosis of Krabbe disease (after our own evaluation with the ExAC server and also in consultation with the Wadsworth Center in NY). All of the samples contained one or more copies of the non-pathogenic variants p.R184C, p.T645A, p.I562T, or p.Y319C.⋯The latter is a common variant in the Southeast Asian population.

**Fig. 2:**
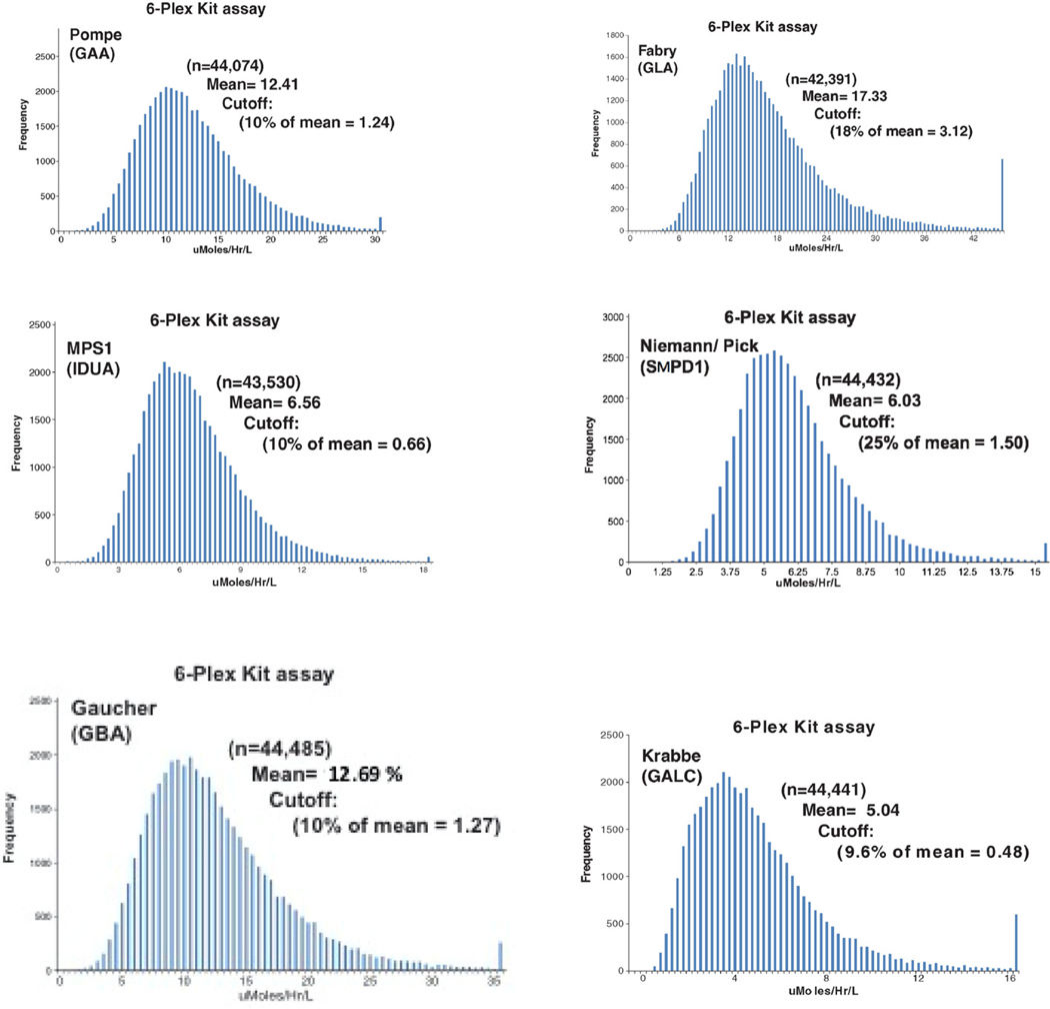
Distribution of 6 enzyme activities in the 6-plex.

The distributions of the 6 enzyme activites across the study population are shown in Fig. 2.

## 4. Discussion

### 4.1. New 6-plex assay

The experimental protocol for the 6-plex assay for Pompe, MPS-I, Niemann-Pick-A/B, Gaucher, Fabry and Krabbe diseases has been simplified from its earlier versions [7,8,16] by using a single assay cocktail with a highly optimized buffer for all enzymes and by shortening the pre-MS/MS workup by elimination of the solid-phase extraction step. Although the 6-plex assay can be performed using liquid chromatography coupled to MS/MS (the method currently being used in the IL state NBS laboratory), many NBS laboratories prefer flow injection (the method being used in NY, NJ, OH, TN, and KY state NBS labs and all 3 NBS labs in Taiwan). The 6-plex has been piloted in the WA NBS laboratory using a single MS/MS instrument. Most NBS laboratories may opt to acquire a backup MS/MS instrument or use the backup instrument already in the laboratory to support NBS analysis of amino acids and acyl carnitines. A recent study has also shown that the same MS/MS instrument can be used for both amino acid/acyl carnitine and lysosomal storage diseases analyses (Poster P-87, APHL Newborn Screening Symposium, 2016, St. Louis, http://www.aphl.org/conferences/Pages/2016-NBSGTS.aspx), and thus smaller NBS laboratories with extra bandwidth on their MS/MS instruments can add lysosomal storage diseases without the acquisition of new equipment.

### 4.2. Study results

For some of the screen positive samples, a clear disease prediction can be made as noted in the Results section. However, since the DBS are de-identified in our study, we cannot be certain of the clinical disease status for many of the positive samples. Even in studies with identified DBS where the children have undergone a clinical examination, a majority fraction of the disease-predicted patients are asymptomatic and may only develop the lysosomal storage disease at a later date (for example see [23]). According to the Policy Statement of the American College of Medical Genetics and Genomics [25], a false positive is defined as a patient who received a screen positive result who does not have disease symptoms. Thus, it seems premature to label patients as positives when they carry mutations predictive of late-onset lysosomal storage disease but have not yet developed symptoms. Nevertheless, it is useful to report the number of such patients. It is for this reason that we focus in this publication only on statements about the number of NBS screen positives.⋯Table 1 gives the number of screen positives for each of the 6 enzyme assays.

### 4.3. Analytical range and comparison of MS/MS versus fluorimetric assays

The analytical range of the assay for an enzyme is defined as the enzyme-dependent assay response with the quality control HIGH sample (typical of a healthy person) divided by the assay response due to all enzyme-independent processes. The rigorous methods used to calculate the analytical ranges for the MS/MS and fluorimetric assays are given in Supplemental material. The analytical range for the MS/MS assay for each of the 6 enzymes are: GAA (88), IDUA (83), GLA (95), GBA (178), SMPD1 (70), and GALC (93) (calculated from Table 5, Data in Brief).⋯The analytical range for the corresponding fluorimetric assay with 4-methylumbelliferyl substrates with a standard plate reader under identical incubation conditions are available for GAA (12) and IDUA (12) [26] and thus are an – 7- to 15-fold lower than for the MS/MS assays. In Supplemental material we show that the analytical ranges for the digital microfluidics fluorimetric assays [27] should be less than the analytical ranges found with a fluorimetric 96-well plate reader.

It is generally expected that an assay with a higher analytical range will be more accurate since the enzyme activity values are measured by “spreading the scores” more. This in turn predicts a lower number of screen positives as the analytical range increases.

NY and MO NBS laboratories have carried out large pilot studies of NBS for Pompe disease, and thus it is useful to compare the results of these studies to the current results. The number of screen positives depends of course on the choice of screen cutoff values. The cutoff values in MO and NY where chosen based on an identical method, to be just above the enzyme activities measured for an identical (lab exchanged) set of DBS that included patients confirmed to have infantile Pompe disease, patients predicted by genotyping to have late onset Pompe disease, and patients containing low enzymatic activity and found to have DNA variations of unknown pathogenicity. As shown in Table 2, the number of Pompe disease screen positives for 100,000 DBS is 20–21 for NY and WA (MS/MS) and 48 for MO (digital microfluidics fluorimetry). For this comparison, we used the same cutoff value in WA as was used in NY (15% of the daily mean). NY uses the same method for Pompe NBS as reported here, the only difference in NY is that the ethyl acetate extract from the Pompe reaction is combined with a separate DBS extract for simultaneous analysis of C26-lysophosphatidylcholine for NBS of X-linked adrenoleukodystrophy. Our cutoff of 10% reported in Table 1 is based on the observation that all the screen positives in the 10–15% range gave only wild type or polymorphism genotypes. It must be noted that the populations used are different in the 3 NBS laboratories, but the results suggest that MS/MS NBS of Pompe disease gives a ~2-fold lower number of screen positives than digital microfluidics fluorimetry.

NY NBS laboratory uses a cutoff of 12% of the daily mean for GALC (Krabbe) and finds a screen positive rate of 18 per 100,000 [24], which compares well to our value of 25 hits with a cutoff of 9.6% of the daily mean. For IDUA (MPS-I) MO NBS laboratory reports 29.1 screen positives per 100,000 [4], and we find 13.6 per 100,000 (Table 1, main text). For GLA (Fabry) MO NBS laboratory reports 62.8 screen positives per 100,000 [4] and we find 18.2 (Table 1, main text). Finally for GBA (Gaucher) MO NBS laboratory reports 11.4 screen positives per 100,000 [4], and we find 6.8 (Table 1, main text).

## Supplementary Material

suppl

## Figures and Tables

**Table 1:** Summary of screen positives for the 6-plex MS/MS assay.

Enz. activity % of daily mean (μmol/h/L)	Genotype	Prediction (ref.)
*MPS-I (IDUA) Cutoff 10%*		
4.2% (0.29)	p.G253D/wt	Carrier
4.0% (0.31)	p.P302T/wt	Carrier
8.6% (0.45)	p.A79T/p.A79T	Low act. variant (ExAC web site)
7.3% (0.48)	p.A79T/p.A79T	Low act. variant (ExAC web site)
7.1% (0.54)	p.G33H/p.G33H	Low act. variant (ExAC web site)
8.3% (0.62)	p.D223N/c.299 + 6C N T	Low act. variant (ExAC web site)
*Pompe (GAA) Cutoff 10%*		
9.2% (1.05)	p.G576S/p.T602I	Probable low activity variant [22]
5.5% (0.56)	c.2168del13ins10/c.2168del13ins10	Affected (see maintext)
*Fabry (GLA) Cutoff 18%*		
17.4 (3.29)	wt/	Unaffected
16.5% (3.00)	wt/	Unaffected
12.5% (2.31)	p.R196K/	Benign variant (ExAC web site)
	p.D66G/	Benign variant (ExAC web site)
17.2% (2.93)	p.K213N/	Affected interferes with splice site
11.5% (2.12)	p.A143T/	Benign variant (unpublished consensus)
17.5% (2.97)	p.L213N/	Affected (see Results section)
11.8% (2.40)	p.R356Q/	Affected found in Fabry registry
*Gaucher (GBA) Cutoff 10%*		
9.8% (1.43)	wt/wt	Unaffected
8.0% (1.14)	p.N370S/wt	Carrier [28]
7.5% (0.97)	p.A309G/p.A309G	Affected [29]
*Niemann-Pick-A/B (SMPD1) Cutoff 25%*		
22.9% (1.18)	wt/wt	Unaffected
25% (1.47)	p.S510F/wt	Low activity variant (ExAC web site)
17.8% (1.04)	p.G508R/p.G508R	Low activity variant (ExAC web site)
23.9% (1.48)	p.P332R/p.G508R	Low activity variant (ExAC web site)
25.0% (1.45)	p.Q21X/p.Q21X	Affected
*Krabbe (GALC) Cutoff 10%*		
9.2% (0.46)	p.T641A/p.I562T	Unaffected (ExAC web site)
9.7% (0.48)	p.I562T/p.T641A	Unaffected (ExAC web site)
9.2% (0.38)	p.T641A/wt	Unaffected (ExAC web site)
7.9% (0.37)	p.T641A/p.I562T	Unaffected (ExAC web site)
8.4% (0.38)	p.R184C/p.T641A	Unaffected (ExAC web site)
7.9% (0.43)	p.R184C/p.Y319C/p.T641A	Unaffected (ExAC web site)
9.0% (0.49)	p.R184C/p.I562T/p.T641A	Unaffected (ExAC web site)
9.3% (0.43)	p.R184C/p.Y319C/p.T641A	Unaffected (ExAC web site)
10% (0.47)	p.R184C/p.T641A	Unaffected (ExAC web site)
8.8% (0.47)	p.R184C/p.Y319C/p.T641A	Unaffected (ExAC web site)
9.7% (0.60)	p.I562T/p.T641A/p.T641A	Unaffected (ExAC web site)

**Table 2:** Number of pompe screen positives for three large studies in WA, NY and MO state NBS laboratories.

NBS Laboratory/method	Analytical range^a^	Screen positives per 100,000 DBS^b^
WA/MS/MS	88	20
NY/MS/MS	66	21
MO/digital microfluidics	<<16.6	48

a Analytical range derived in Supplemental material (unpublished data from J. Orsini, Wadsworth Center, NY used to calculate the value in NY).

b The value in WA is extrapolated from ~43,000 DBS (this study), the value in MO is from 175,000 DBS [23], and the value in NY is from ~330,000 DBS (courtesy of J. Orsini, Wadsworth Ctr, Albany, NY).

## Appendix A. Supplementary data

Supplementary data to this article can be found online at http://dx.doi.org/10.1016/j.ymgme.2016.05.015.

## Abbreviations

DBS: Dried blood spot on a newborn screening card
GAA: Acid α-glucosidase
GALC: Galactocerebrosidase
GBA: Glucocerebrosidase
GLA: α-Galactosidase A
IDUA: α-Iduronidase
MPS-I: Mucopolysaccharidosis-I
MS/MS: Tandem mass spectrometry
MRM: Multiple reaction monitoring
NBS: Newborn screening
SMPD1: Sphingomyelin phosphodiesterase-1
