# Supplementary material for: Pilot study of newborn screening for six lysosomal storage diseases using Tandem Mass Spectrometry
Source: Mol Genet Metab. Author manuscript; Available in PMC 2017 Feb 20. (PMC5318163; doi:10.1016/j.ymgme.2016.05.015)
Supplement: suppl [file NIHMS849461-supplement-suppl.pdf]

Supplemental Material

Table of Contents.

1. Calculation of the Analytical Range
2. Buffer optimization
3. References.

1. Calculation of the Analytical Range.

MS/MS. The enzymatic activity obtained for the quality control HIGH sample is obtained by multiplying the P/IS ratio (where P is MRM ion counts obtained by integration of the MRM detector response versus time over the flow injection peak profile, and IS is the corresponding value for the internal standard MRM peak) by the micromoles of internal standard in the assay well then dividing by the incubation time and the volume (in liters) of blood in each assay (taken as 3.2×10^{-6} L for the 3.0 mm DBS punch). The corresponding enzyme-independent assay response was obtained when a filter plate (no blood) punch was incubated with the assay cocktail and processed as for the DBS sample. This blank activity contains contributions from the following: 1) Any product present as a trace impurity in the substrate or internal standard added to the assay; 2) Any non-enzymatic breakdown of substrate to product generated during the incubation in assay buffer; 3) Any assay signal coming from non-product components of the sample injected into the MS/MS (i.e. blood components, filter paper, buffer, workup solvents, flow-injection solvent, plates); 4) Any product generated from substrate breakdown in the heated electrospray ionization source of the MS/MS instrument. The analytical range is calculated using the equation below, and values for the 6 enzymes are given in the main text.

Analytical Range =

[(activity of quality control HIGH assay)- (activity of the enzyme-independent assay)] / (activity of the enzyme-independent assay)

Blank factor 1 above was measured by analyzing substrate and internal standard by liquid chromatography-MS/MS under conditions in which substrate and product/internal standard are baseline separated during chromatography. Blank factor 2 was measured by running the incubated assay with a filter paper punch (no blood), and then measuring the amount of product by liquid chromatography-MS/MS. Blank factor 3 was measured by running the incubated assay with a DBS punch but in the absence of substrate and analyzing for product by liquid chromatography-MS/MS. Blank factor 4 was obtained by flow injection-MS/MS on a substrate/internal standard mixture. Note that blank factors 1-3 were obtained from liquid-chromatography-MS/MS rather than flow injection-MS/MS. Since substrate and product are baseline separated during liquid chromatography, the product MRM signal is devoid of any signal coming from in-source breakdown of substrate. These studies show that by far the major contribution to the enzyme-independent blank is blank factor 4 (blank factors 1-3 account for < 5% of blank factor 4). This study shows that the analytical range is properly calculated from the enzyme activity measured for the incubated assay with DBS and substrate and the enzyme activity measured for the incubated assay with filter paper only (no blood) and substrate. This method of calculating the analytical range has been previously reported [1].

Fluorimetry. To calculate the analytical range for fluorimetric assays with 4-methylumbelliferyl substrates, a different method is needed. Components of blood (hemoglobin, etc.) substantially quench the fluorescence and have to be taken in to account. Both the blood sample and the substrate may display intrinsic fluorescence. Finally, the substrate may contain a trace of product as an impurity and also suffer non-enzymatic breakdown to product in the absence of enzyme.

As described previously [1], we prepared two samples to measure the blank. Tube A contains DBS punch and a volume of assay buffer equal to half the volume in the complete assay. Tube B contains substrate at twice the concentration as in the complete assay and in a volume of buffer equal to that in the complete assay. Both tubes are incubated as for the complete assay. After incubation, half the volume of tube B is transferred to tube A, and the mixture is immediately quenched and processed for fluorimetry as for the complete assay. In this way the blank will display fluorescence from the following processes: 1) from the substrate itself, 2) from the buffer, 3) from the blood; 4) from product present in the substrate as an impurity; 5) from product generated non-enzymatically from substrate. Also quenching of the fluorescence by blood will occur to the same extent as for the complete assay. Studies show that the predominant factor contributing to the blank fluorescence is the intrinsic fluorescence of the substrate itself. The deprotonated form of the 4-methylumbelliferone product is ~1000-fold more fluorescent per mole than the substrate, but since only ~1% of the substrate is converted to product by the small amount of enzyme in the DBS, the increase in fluorescence is on the order of 10-fold [1].

To estimate the analytical range for digital microfluidics fluorimetry (the values have not been reported), we note that the intrinsic fluorescence of the 4-methylumbelliferyl substrate occurs for all fluorimetric methods where the fluorescence of the product, 4-methylumbelliferone (or a derivative) is measured. Quenching by blood components is shown to be less for digital microfluidics presumably because the optical pathlength in the microdroplets is much smaller than in a 96-well plate reader [2]. But since the analytical range is limited by the intrinsic fluorescence of the substrate, the blood quenching does not significantly contribute to the analytical range. The incubation times for the digital microfluidics assays for Pompe, Fabry, and MPS-I are in the 1-3 hr range [2] compared to the overnight incubations done for the MS/MS and the plate reader fluorimetric assays. Thus the analytical ranges for the digital microfluidics assays are expected to be less than the values in the main text.

2. Buffer Optimization.

Buffer conditions were optimized by a 5-factor, 3-level design experiment to optimize pH and buffer concentration, as well as the concentration of sodium taurocholate, sodium oleate, and zinc chloride. Optimization of the inhibitor concentrations for acarbose, *N*-acetylglucosamine and *D*-saccharic acid 1,4-lactone was performed last and included confirmed inhibitor-specific disease-positive patients along with presumed healthy neonatal samples. Three different concentrations were screened for each inhibitor using a one-factor-at-a-time approach, and the optimal amount was determined by maximizing the resolution between the healthy neonatal enzyme activity and that of the confirmed disease-positive sample.

3. References.

[1] A.B. Kumar; Z. Spacil; S. Masi; F. Ghomashchi; N. Chennamaneni; M. Ito; C.R. Scott; F. Turecek; M.H. Gelb, Tandem Mass Spectrometry Has a Higher Analytic Range than Fluorescence Assays of Lysosomal Enzymes: Application to Newborn Screening and Diagnosis of Mucopolysaccharidosis Types II, IVA and VI, Clin. Chem.(61) (2015) 1363.

[2] R.S. Sista; A.E. Eckhardt; T. Wang; C. Grahman; J.L. Rouse; S.M. Norton; V. Srinivasan; M.G. Pollack; A.A. Tolun; D. Bali; D.S. Millington; V.K. Pamula, Digital microfluidic platform for multiplexing enzyme assays: implications for lysosomal storage disease screening in newborns,Clin. Chem. 57 (2011) 1444.
